# Editorial

**Published:** 1901-04-15

**Authors:** 


### EDITORIAL.

The following letter explains itself and carries its own inference. And we trust the Examiners may have the courage of their convictions and stand firmly for a high standard of attainment not only for the so-called practical or technical department of our profession, but for the theorectical as well. This will insure a proper and adequate army and navy dental service, and demonstrate the propriety of such service. It will also do away with preferment, because of political influence. We have no doubt, if the plan of selection were put on any other than an educational basis, great injustice would be done to the service, and our profession would be discredited.

Editor Dental Register.

*Dear Sir:*—By permission of the Surgeon General, I have the honor to inform you that the follownig named gentlemen have successfully passed the examination before this Board, and have received their appointments as Contract Dental Surgeons, U. S. Army:

Siebert Davis Boak, of Martinsburg, West Va.; Edward Clarence Lauderdale, of Naples, N. Y.

These gentlemen have been ordered to report for duty at San Francisco, Cal., April 15th, for service in the Philippines.

At the present writing there have been fourteen gentlemen ordered before this Board by the Surgeon General for examination. Only two, as you see by the above report, have successfully passed the examination. The Board has been disappointed in the professional qualifications of most of the young men who have presented themselves. The examination does not cover any subjects which have not been taught in our best dental schools, and the Board believes that the questions submitted in the examination have been of a practical nature, and eminently fair. It is to be hoped therefore that our dental schools will not recommend any young men to come before this Board who are not thoroughly well qualified, theoretically and practically, in all of the branches comprising the curriculum of our best dental schools.

It will be the pleasure of the Examining Board of Dental Surgeons for the Army to keep the profession posted as to its work through the various dental journals.

Very respectfully,

JOHN S. MARSHALL,

*President Examining Board of Dental Surgeons.*

WASHINGTON, D. C., March 16, 1901.

---

THE editor of the *American Dentist* in the February issue comments at considerable length on the deplorable lack of legal restraint placed upon unqualified and disreputable practitioners of dentistry in the State of Ohio. According to his statement the State is over-run with professional rascals and incompetents, and there seems at present no prospect of change for the better. Several attempts have been made to amend the present statute so that a higher standard of qualification might be required, and that well-defined penalties should be required for illegal or incompetent practice. But because of the numerical or political strength, in its organized capacity, of the "degenerate" part of the profession, no adequate legislation seems possible. We are not inclined to look upon the matter so pessimistically as the editor of the *American Dentist*, and yet we do know that there is truth in the statement, but we are confident the present Board of Exainmers is doing all it can to protect the people from incompetent practitioners. The editor of the *Ohio Dental Journal* is in a position to make an official declaration on this subject that would reveal the truth ; and should it be as bad as represented it will assuredly stimulate the truly professional to make a concerted movement to have adequate laws enacted for the suppression of quackery and the protection of the public. Dr. Bethel, it is up to you.

---

A NEWSPAPER report from McKeesport, Pa., relates that a man thirty years of age died from heart failure, brought on by a hypodermic injection of cocain solution used for the extraction of a number of teeth several months previous to the fatality. As usual in such reports there are no particulars upon which to base an opinion, but it seems incredible that cocain itself should be responsible for such a result. It is possible that the hypodermic injection may have been improperly made, in view of the existing conditions, resulting in systemic inoculation from the incisted pus in the alveolus. An injection of distilled water, used under similar conditions, would probably have produced the same result. In our anxiety to secure painless operations, it may be possible that we are making the mistake of taking into account the pharmacological properties only of drugs, to the exclusion of their therapeutic application. We can not dispense with local anesthetics, and especially with so valuable a drug as cocain. But a drug of such power ought to be used with great discretion. And a person who is unwilling to take the trouble to carefully study his cases when using any drug, however benign in action it may be, ought not to be surprised at the unexpected symptoms which follow as a consequence of careless manipulation.

---

We have received a circular announcing the organization of a dental college at Manila, of which Dr. Louis Ottofy, formerly of Chicago, is Dean. The reasons for its existence is given as follows: First, to equip the natives for the practice of a profession much needed; second, to permit such Americans as may have started the study in the United States to complete the course in the Philippines; third, to furnish opportunity for such Americans as have come out to the Philippines for other purposes to acquire a professional education without returning home. We, of course, do not know the situation, and consequently can neither justify nor deny these statements. But it would seem that there could be no urgent necessity for the organization of a dental college at this time. The natives are not prepared for it, and we doubt if adequate facilities can be had for the proper conduct of a creditable institution at present.

---

The *American Journal of Dental Science* has ceased publication. It was the first dental journal ever published. It was first issued in 1839, and, with the exception of a lapse of seven years from 1860 to 1867, it has been published continuously until the July (1900) number. Its early history was one of exceedingly important moment to the welfare of the profession. Of late years it has not been an important factor in dental literature. Its suspension leaves the DENTAL REGISTER as the oldest dental journal published.

---

DR. A. E. WEBSTER, of the Royal College of Surgeons, of Toronto, is the new editor of the *Dominion Dental Journal*. He succeeds Dr. W. G. Beers, recently deceased. We predict prosperity and a brilliant future for this journal under Dr. Webster's management. He is young, vigorous, and full of professional spirit.

